# Association between dietary index for gut microbiota and chronic kidney disease: A cross-sectional study from U.S. population

**DOI:** 10.1016/j.pmedr.2025.103060

**Published:** 2025-04-08

**Authors:** Xuanzhen Zhou, Chengxiao Jiang, Baiyang Song, Shuben Sun, Zejun Yan

**Affiliations:** a Department of Urology, Ningbo Clinical Research Center for Urological Disease, Zhejiang Engineering Research Center of Innovative technologies and diagnostic and therapeutic equipment for urinary system diseases, The First Affiliated Hospital of Ningbo University, Ningbo, China; b Department of Urology, Xiangshan Frist People's Hospital Medical and Health Group, Xiangshan, China

**Keywords:** Dietary index, Gut microbiota, Chronic kidney disease, DI-GM, NHANES

## Abstract

**Objective:** Emerging evidence suggests that diet modulates gut microbiota, which in turn influences chronic kidney disease (CKD) progression. This study investigates the association between the newly proposed Dietary Index for Gut Microbiota (DI-GM) and the prevalence and prognosis of CKD.

**Methods:** This cross-sectional study analyzed data from the U.S. National Health and Nutrition Examination Survey 2007–2020. DI-GM scores were calculated based on dietary intake of 14 food components, categorized as beneficial or unfavorable. Weighted linear regression model, logistic regression model, and restricted cubic spline analysis were used to assess the associations of DI-GM with CKD.

**Results:** The prevalence of CKD among 28,512 participants was 17.4 %. Higher DI-GM was negatively associated with CKD prevalence (OR = 0.967, 95 %CI: 0.939–0.995, *p* = 0.026) and with very high-risk prognosis (OR = 0.877, 95 %CI: 0.821–0.937, *p* < 0.001). Beneficial DI-GM components were significantly associated with lower CKD risk (OR = 0.928, 95 %CI: 0.892–0.966, *p* < 0.001), while no significant association was observed for unfavorable components. Higher DI-GM and beneficial DI-GM levels were linearly associated with improved CKD prognosis (*p* for trend <0.001). Coffee and fiber were primary contributors to both the prevalence and prognosis of CKD, while whole grains primarily impacted its prognosis.

**Conclusions:** Higher DI-GM, driven by beneficial dietary components, is associated with reduced CKD prevalence and improved prognosis. These findings suggest that promoting beneficial dietary patterns to enhance gut microbiota may play a pivotal role in CKD management.

## Highlights

• Dietary index for gut microbiota linked to chronic kidney disease prevalence.
• Higher dietary index for gut microbiota improves prognosis in chronic kidney disease.
• Beneficial components of dietary index for gut microbiota play a key role.
• Dietary interventions promoting gut health may help prevent chronic kidney disease.

## 1 Introduction

Chronic kidney disease (CKD) is a progressively debilitating condition characterized by the irreversible loss of nephron function, ultimately leading to end-stage renal disease. The global prevalence of CKD has surged in recent decades, affecting approximately 9.1 % of the world's population and imposing a significant burden on healthcare systems worldwide (Global, regional, and national burden of chronic kidney disease, 1990-2017). An estimated 37 million U.S. adults have CKD, affecting more than one in seven individuals (*Chronic Kidney Disease in the United States. Centers for Disease Control*, 2025). Despite its widespread impact, CKD often remains undiagnosed in its early stages due to the absence of apparent symptoms. More strikingly, nine in ten adults are unaware of their CKD, and half of those with significantly reduced kidney function not on dialysis remain unaware of their condition (*Chronic Kidney Disease in the United States. Centers for Disease Control*, 2025). Current treatments are largely limited to slowing disease progression and managing symptoms through therapies such as renin-angiotensin system blockade, highlighting the pressing need for novel therapeutic approaches (Ruiz-Ortega et al., 2020).

Recent studies have increasingly focused on the role of diet in the prevention and progression of CKD. Dietary interventions, such as the Dietary Approaches to Stop Hypertension and plant-based diets, have demonstrated potential benefits in managing CKD by alleviating oxidative stress, inflammation, and metabolic derangements (Couch et al., 2024; Naber and Purohit, 2021). Notably, dietary patterns rich in antioxidants—measured through indices like the Composite Dietary Antioxidant Index—have been shown to be associated with a reduced prevalence of CKD and lower rates of all-cause mortality. Emerging evidence suggests that dietary factors significantly influence the gut microbiome, which, in turn, modulates metabolic regulation, immune function, systemic inflammation, and kidney function (Culp et al., 2024; Delzenne et al., 2025; Ko et al., 2020; Ranganathan and Anteyi, 2022). Moreover, accumulating evidence has highlighted the crucial role of the gut-kidney axis in CKD. Gut dysbiosis, a common occurrence in CKD, is characterized by an imbalance in the gut microbiota, leading to the generation of gut-derived uremic toxins that exacerbate kidney damage (Huang and Chen, 2024). This underscores the importance of gut health in CKD management.

The Dietary Index for Gut Microbiota (DI-GM) is a recently proposed metric that reflects the diversity and quality of gut microbiota through dietary patterns (Kase et al., 2024). Studies have demonstrated a link between a higher DI-GM and improved mental health outcomes, such as reduced prevalence of depression, highlighting the relevance of diet in modulating gut microbiota and its systemic effects (Zhang et al., 2024). Despite growing evidence linking CKD to gut microbiota alterations, no research has yet explored the association between DI-GM and CKD. To address this gap, we analyzed data from the National Health and Nutrition Examination Surveys (NHANES) to evaluate the association of DI-GM with CKD and its prognosis.

## 2 Methods

### 2.1 Data source

The NHANES is a comprehensive, multistage survey conducted by the National Center for Health Statistics, biennially captures nationally representative data of the U.S. non-institutionalized civilian population (Chen et al., 2020). The protocol of NHANES was approved by the National Center for Health Statistics Institutional Review Board (Protocol #2005–06, #2011–17, #2018–01). All participant provided informed written consent prior to participation. Therefore, no additional ethical review was required for this study. Detailed information on study design, data collection, and measurement protocols is publicly available at https://www.cdc.gov/nchs/nhanes/index.html.

### 2.2 Population selection

**Fig. 1:**
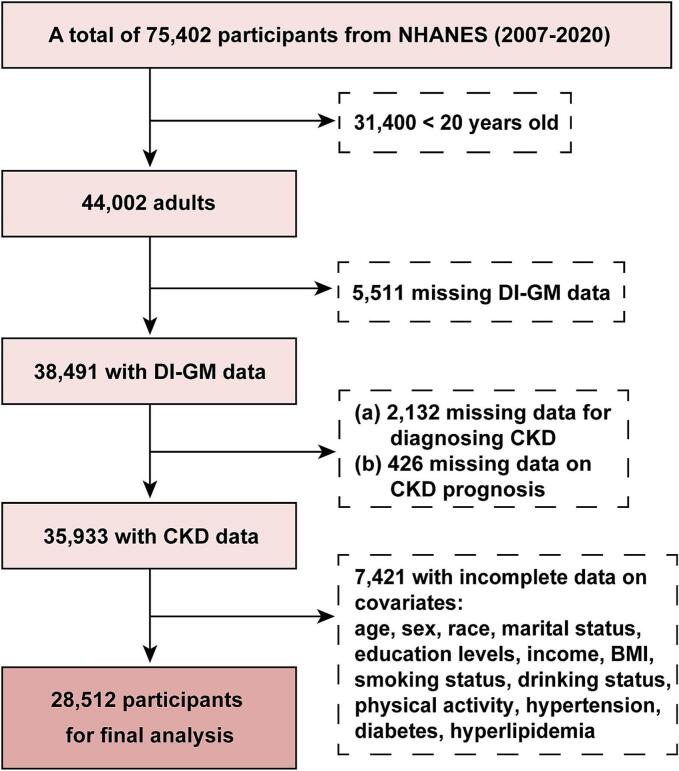
Flowchart of participants selection: NHANES 2007–2020 for U.S. adults.

In this cross-sectional study, we analyzed data from seven cycles (2007–2020) during which DI-GM information was available. Initially, 75,402 participants were identified from the datasets. Then, we excluded participants based on the following criteria: age under 20 years, missing DI-GM data, missing data for diagnosing CKD, missing data on CKD prognosis, and incomplete covariate data. Finally, 28,512 participants were included in the analysis. The detailed screening process is illustrated in Fig. 1.

### 2.3 Assessment of DI-GM

As outlined in previous studies, 14 food items or nutrients were identified as components of DI-GM (Kase et al., 2024; Zhang et al., 2024). Beneficial components included avocado, broccoli, chickpeas, coffee, cranberries, fermented dairy, fiber, green tea, soybean, and whole grains. Adverse components consisted of refined grains, red meat, processed meat, and a high-fat diet (≥ 40 % of energy from fat). Dietary data from a 24-h recall were used to calculate the DI-GM. For beneficial DI-GM, a score of 1 was assigned if consumption was ≥ the sex-specific median; otherwise, a score of 0 was given. For unfavorable DI-GM, a score of 0 was assigned if consumption was ≥ the sex-specific median or ≥ 40 % (for a high-fat diet); otherwise, a score of 1 was assigned.

### 2.4 Definition of CKD

Since most participants had only a single measurement in the survey, and the timing of the second urine sample collection varied across selected NHANES cycles or subsamples, we used a one-time urinary albumin-to-creatinine ratio (UACR) as a substitute for 24-h persistent proteinuria to minimize bias (Chen et al., 2023; Murphy et al., 2016). The Chronic Kidney Disease Epidemiology Collaboration equation was used to estimate glomerular filtration rate (eGFR) (Levey et al., 2009). According to the KDIGO, 2024 Clinical Practice Guideline, CKD is defined as a UACR ≥30 mg/g and/or an eGFR <60 mL/min/1.73 m^2^ (KDIGO, 2024).

CKD prognosis is classified based on eGFR category (G1–G5) and albuminuria category (A1–A3) (KDIGO, 2024). According to the guidelines, CKD is divided into four prognostic categories: low risk (eGFR levels G1 or G2 with UACR A1 [G1/2-A1]; if no other markers of kidney disease are present, CKD is not diagnosed), moderate risk (G2–A1, G1/2–A2), high risk (G3b–A1, G3a–A2, G1/2–A3), and very high risk (G3a–A3, G3b–A2/3, G4, G5).

### 2.5 Covariates

Data on demographics, lifestyles, and comorbidities were collected, including age, sex, race/ethnicity, marital status, education level, household income, body mass index, smoking status, alcohol consumption, physical activity, hypertension, diabetes mellitus, and hyperlipidemia. No significant multicollinearity was found by calculating the variance inflation factor for each variable (all values <5). Detailed information on the classification of household income (Zhang et al., 2021), smoking status (Xie et al., 2024), drinking status (Chen et al., 2016), physical activity, hypertension (La et al., 2024b), diabetes mellitus (*Diabetes Care*, 2025), and hyperlipidemia (La et al., 2024a) is provided in Supplementary Table S1.

### 2.6 Statistical analysis

Continuous variables were presented as medians (interquartile range) and analyzed using the weighted Wilcoxon rank-sum test. Categorical variables were presented as frequencies with percentages (%) and analyzed using the weighted chi-squared test. Multivariable linear regression model was performed to investigate the association between DI-GM and the key markers of kidney function, with results presented as *β* along with 95 % confidence intervals (CI) and *p*-values. Multivariable logistic regression model was performed to investigate the association between DI-GM and CKD. The results were reported as odds ratios (OR) with 95 % CI and *p*-values. The DI-GM was categorized into groups based on pre-established classifications from previous studies (Zhang et al., 2024), and *p*-values for trends were calculated. Three models were used: Model 1 was unadjusted for covariates, Model 2 adjusted for key demographic variables, and Model 3 adjusted for all covariates. Restricted cubic spline model was used to graphically represent the dose-response relationship between DI-GM and CKD. To identify the association between specific components of DI-GM and the prevalence and prognosis of CKD, we further adjusted for all components of DI-GM based on Model 3. A two-sided *p*-value <0.05 was considered statistically significant. Appropriate weighting methodologies were employed in accordance with NHANES guidelines (Johnson et al., 2013). All statistical analyses were performed using R software version 4.3.3.

## 3 Results

### 3.1 Baseline characteristics

The weighted baseline characteristics of participants are presented in Table 1. The overall sample consisted of 28,512 participants, with a mean age of 47(33,60) years, and a gender distribution of 49.33 % male and 50.67 % female. The prevalence of CKD is 17.4 % among these participants. Compared to participants without CKD, those with CKD were older, more likely to be female, Non-Hispanic White, or Black, and were more frequently widowed, divorced, or separated. They also had lower education levels, lower household income, were more often former smokers, and more likely to be never or former drinkers. Participants with CKD engaged in less physical activity, had a higher prevalence of hypertension, diabetes mellitus, hyperlipidemia, and had a higher body mass index. However, no statistically significant differences were found between the groups in terms of DI-GM or DI-GM group.

**Table 1:** Baseline characteristics stratified by chronic kidney disease among U.S. adults from NHANES 2007–2020.

Variable	Overall *n* = 28,512	Non-CKD *n* = 23,561	CKD *n* = 4951	*P*-value
Age, years (IQR)	47 (33, 60)	45 (32, 57)	64 (49, 75)	< 0.001
Age group, n (%)				< 0.001
20–39	9565 (36.3)	8977 (39.8)	588 (15.2)	
40–59	9639 (37.9)	8490 (39.7)	1149 (26.9)	
60+	9308 (25.7)	6094 (20.6)	3214 (57.9)	
Sex, n (%)				< 0.001
Female	14,341 (50.7)	11,792 (49.8)	2549 (56.2)	
Male	14,171 (49.3)	11,769 (50.2)	2402 (43.8)	
Race/ethnicity, n (%)				< 0.001
Mexican American	4083 (8.1)	3466 (8.3)	617 (6.8)	
Non-Hispanic Black	5939 (10.0)	4832 (9.8)	1107 (11.3)	
Non-Hispanic White	12,325 (69.2)	9943 (68.8)	2382 (71.3)	
Other	6165 (12.8)	5320 (13.1)	845 (10.6)	
Marital status, n (%)				< 0.001
Never married	5266 (18.2)	4725 (19.4)	541 (10.8)	
Married/Living with Partner	17,023 (63.8)	14,292 (64.4)	2731 (60.0)	
Widowed/Divorced/Separated	6223 (18.0)	4544 (16.2)	1679 (29.2)	
Education levels, n (%)				< 0.001
Less than high school	6058 (13.6)	4699 (12.7)	1359 (19.0)	
High school or equivalent	6496 (23.1)	5274 (22.6)	1222 (26.2)	
College or above	15,958 (63.3)	13,588 (64.7)	2370 (54.8)	
Household income, n (%)				< 0.001
Low	5814 (13.5)	4719 (13.2)	1095 (15.5)	
Middle	15,003 (47.8)	12,198 (47.0)	2805 (53.2)	
High	7695 (38.7)	6644 (39.8)	1051 (31.3)	
Smoking status, n (%)				< 0.001
Never	15,865 (56.0)	13,377 (56.7)	2488 (51.7)	
Former	6919 (25.1)	5307 (23.8)	1612 (33.1)	
Now	5728 (18.9)	4877 (19.5)	851 (15.2)	
Drinking status, n (%)				< 0.001
Never	3694 (9.9)	2888 (9.3)	806 (13.7)	
Former	3694 (10.9)	2663 (9.8)	1031 (18.3)	
Now	21,124 (79.2)	18,010 (81.0)	3114 (68.0)	
Physical activity, n (%)				< 0.001
None	15,872 (51.7)	12,717 (50.4)	3155 (59.9)	
Moderate	6442 (25.0)	5399 (25.2)	1043 (23.8)	
Vigorous	6198 (23.3)	5445 (24.4)	753 (16.3)	
Hypertension, n (%)				< 0.001
No	16,352 (62.4)	14,970 (67.2)	1382 (32.6)	
Yes	12,160 (37.6)	8591 (32.8)	3569 (67.4)	
Diabetes mellitus, n (%)				< 0.001
No	23,149 (85.8)	20,240 (89.2)	2909 (64.5)	
Yes	5363 (14.2)	3321 (10.8)	2042 (35.5)	
Hyperlipidemia, n (%)				< 0.001
No	8258 (29.9)	7400 (32.0)	858 (17.3)	
Yes	20,254 (70.1)	16,161 (68.1)	4093 (82.7)	
Body mass index, kg/m^2^ (IQR)	28.10 (24.30, 32.80)	27.90 (24.20, 32.50)	29.44 (25.20, 34.60)	< 0.001
DI-GM (IQR)	5.00 (4.00, 6.00)	5.00 (4.00, 6.00)	5.00 (4.00, 6.00)	0.2
DI-GM group, n (%)				0.53
0–3	6998 (22.9)	5712 (22.7)	1286 (24.1)	
4	7006 (23.5)	5788 (23.6)	1218 (23.1)	
5	6573 (23.4)	5439 (23.4)	1134 (23.5)	
6+	7935 (30.1)	6622 (30.2)	1313 (29.4)	
UACR, mg/g (IQR)	6.53 (4.36, 11.52)	5.96 (4.18, 9.14)	41.85 (12.81, 93.29)	< 0.001
eGFR, mL/min/1.73 m^2^ (IQR)	95.99 (80.88, 109.97)	97.84 (84.59, 110.98)	65.85 (52.82, 96.87)	< 0.001

The data are presented as frequencies (%) or median (IQR). All estimates were obtained from complex survey designs, *p*-values were obtained from *t*-tests or Kruskal-Wallis tests for continuous variables based on their distribution, and from Chi-square tests for categorical variables. IQR interquartile range, CKD chronic kidney disease, DI-GM dietary index for gut microbiota, NHANES National Health and Nutrition Examination Survey, UACR urinary albumin-to-creatinine ratio, eGFR estimate glomerular filtration rate.

### 3.2 Association between DI-GM and CKD prevalence

As described in Table 2, weighted multivariable logistic regression revealed the negative associations of continuous DI-GM and DI-GM quartiles with the prevalence of CKD. After full adjustment (Model 3), each unit increase in DI-GM was associated with a 3.3 % decrease in the prevalence of CKD (OR = 0.97, 95 %CI: 0.94–0.99, *p* = 0.026). Compared with the lowest DI-GM group (0–3), the highest group (≥ 6) was associated with a 12.7 % decrease in the prevalence of CKD (OR = 0.87, 95 %CI: 0.77–0.99, *p* = 0.039). However, no significant linear trend was observed across DI-GM groups (*p* for trend = 0.393). After mutual adjustment of DI-GM components, a significant negative association was observed between beneficial DI-GM and CKD (OR = 0.93, 95 %CI: 0.89–0.97, *p* < 0.001), while no significant association was found between unfavorable DI-GM and CKD (OR = 1.03, 95 %CI: 0.99–1.07, *p* = 0.144). In addition, we explored the relationship between DI-GM and two key markers of kidney function, eGFR and UACR (Supplementary Table S2).

**Table 2:** Association between dietary index for gut microbiota and chronic kidney disease in U.S. adults from NHANES 2007–2020.

	Model 1	Model 2	Model 3
	OR (95 %CI)	OR (95 %CI)	OR (95 %CI)
Prevalence			
DI-GM	0.98 (0.96, 1.01)	0.93 (0.90, 0.96)	0.97 (0.94, 0.99)
DI-GM group			
0–3	1.00	1.00	1.00
4	0.92 (0.82, 1.04)	0.9 (0.79, 1.02)	0.95 (0.84, 1.08)
5	0.95 (0.83, 1.08)	0.84 (0.73, 0.96)	0.97 (0.85, 1.11)
≥6	0.92 (0.82, 1.03)	0.73 (0.64, 0.83)	0.87 (0.77, 0.99)
*P* for trend	0.483	0.523	0.393
Beneficial DI-GM	0.93 (0.90, 0.96)	0.89 (0.86, 0.92)⁎	0.93 (0.89, 0.97)⁎
Unfavorable DI-GM	1.08 (1.04, 1.13)	0.99 (0.96, 1.03)#	1.03 (0.99, 1.07)#
Prognosis at very high risk			
DI-GM	0.88 (0.82, 0.94)	0.83 (0.78, 0.89)	0.88 (0.82, 0.94)
DI-GM group			
0–3	1.00	1.00	1.00
4	0.71 (0.54, 0.93)	0.66 (0.50, 0.87)	0.70 (0.53, 0.93)
5	0.77 (0.57, 1.05)	0.68 (0.49, 0.95)	0.83 (0.59, 1.16)
≥6	0.56 (0.43, 0.74)	0.44 (0.34, 0.59)	0.55 (0.41, 0.73)
*P* for trend	0.063	0.034	0.015

All estimates were obtained from complex survey designs, *p* for trend was obtained from multivariable logistic regression analysis.

Model 1: unadjusted.

Model 2: adjusted for age, sex, race/ethnicity.

Model 3: adjusted for age, sex, race/ethnicity, marital status, household income, education level, body mass index, smoking status, drinking status, physical activity, hypertension, diabetes mellitus, hyperlipidemia.

DI-GM dietary index for gut microbiota, NHANES National Health and Nutrition Examination Survey.

⁎ Further adjusted for unfavorable DI-GM.

# Further adjusted for beneficial DI-GM.

### 3.3 Association between DI-GM and CKD prognosis

**Supplementary Figure S1:**
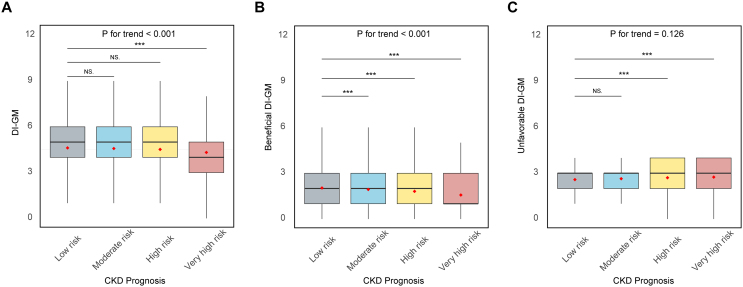
Association between dietary index for gut microbiota (DI-GM) and chronic kidney disease prognosis in U.S. adults from NHANES 2007–2020. (A) DI-GM, (B) beneficial DI-GM, and (C) unfavorable DI-GM. (The Kruskal-Wallis test was used to evaluate differences in DI-GM distributions among CKD prognosis classifications. The Cochran-Armitage trend test was used to assess linear trends in DI-GM as risk progressed. ^⁎⁎⁎^*p* < 0.001, ^NS.^*p* > 0.05, mutual adjusted for DI-GM components based on Model 3)

As shown in Supplementary Figure S1, compared to participants with a low risk of CKD prognosis, those with a very high risk prognosis had significantly lower levels of DI-GM and beneficial DI-GM (both *p* < 0.001) but higher levels of unfavorable DI-GM (*p* < 0.001). As CKD prognosis risk increased, levels of DI-GM and beneficial DI-GM demonstrated significant decreasing linear trends (both *p* for trend <0.001). However, no significant linear trend was observed for the association between unfavorable DI-GM and CKD prognosis (*p* for trend = 0.126). Given the lack of significant differences in DI-GM levels between participants with low, moderate, or high risk prognosis, we further assessed the association between DI-GM and CKD at very high risk prognosis.

As described in Table 2, weighted multivariable logistic regression showed the negative associations of continuous DI-GM and DI-GM quartiles with the prevalence of CKD at a very high risk prognosis. In the Model 3, each unit increase in DI-GM was associated with a 12.3 % decrease in the prevalence of CKD at a very high risk prognosis (OR = 0.88, 95 %CI: 0.82–0.94, *p* < 0.001). Compared with the lowest DI-GM group, the highest group was associated with a 45.3 % decrease in the prevalence of CKD at a very high risk prognosis (OR = 0.55, 95 %CI: 0.41–0.73, *p* < 0.001), with a significant linear trend (*p* for trend = 0.015).

### 3.4 Dose-response relationship

**Fig. 2:**
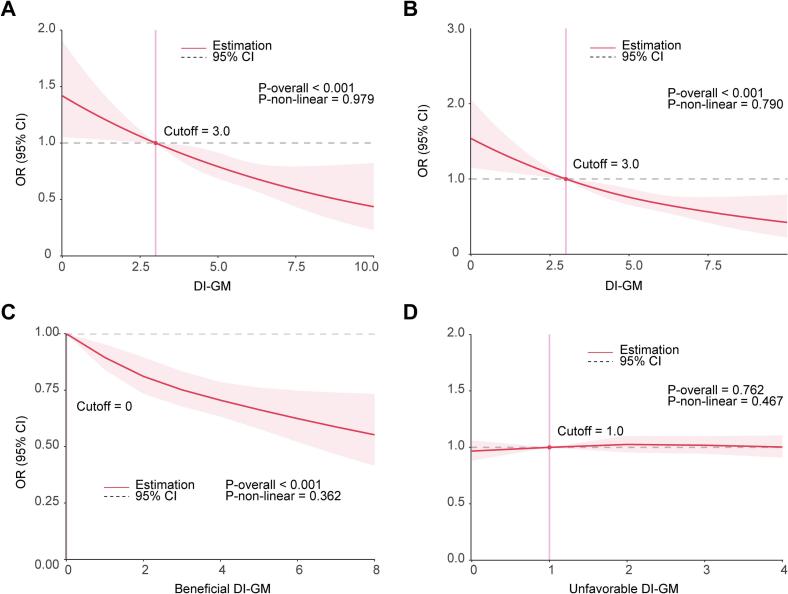
Dose-response relationship between dietary index for gut microbiota (DI-GM) and chronic kidney disease (CKD) in U.S. adults from NHANES 2007–2020. (A) DI-GM and CKD prevalence, (B) DI-GM and CKD prognosis, (C) beneficial DI-GM and CKD prevalence, (D) unfavorable DI-GM and CKD prevalence.

The dose-response relationship between DI-GM and CKD was shown in Fig. 2. After full adjustment, the restricted cubic spline analysis showed a linear association between DI-GM and CKD (*p*-overall <0.001, *p*-non-linear = 0.979). Similarly, a linear association was observed between DI-GM and CKD with a very high-risk prognosis (*p*-overall <0.001, *p*-non-linear = 0.79). After full adjustment and mutual adjustment of DI-GM components, a negative linear association was observed between beneficial DI-GM and CKD (*p*-overall <0.001, *p*-non-linear = 0.362), while no association was found between unfavorable DI-GM and CKD (*p*-overall = 0.762, *p*-non-linear = 0.467).

### 3.5 Association between specific components of DI-GM and CKD

To identify which specific components of DI-GM have a more significant impact on outcomes, we further adjusted all components of DI-GM based on Model 3 (Supplementary Table S3). We found that coffee (OR = 0.83, 95 %CI: 0.75–0.92, *p* = 0.001) and fiber (OR = 0.83, 95 %CI: 0.75–0.91, *p* < 0.001) were independently associated with CKD prevalence. Additionally, coffee (OR = 0.73, 95 %CI: 0.57–0.93, *p* = 0.014), fiber (OR = 0.58, 95 %CI: 0.46–0.74, *p* < 0.001), and whole grains (OR = 0.72, 95 %CI: 0.56–0.94, *p* = 0.018) were independently associated with CKD at very high-risk prognosis.

## 4 Discussion

To our knowledge, this is the first study to investigate DI-GM levels in populations with and without CKD. This study demonstrates a linear negative association between DI-GM and both the prevalence and prognosis of CKD. Notably, beneficial DI-GM was significantly associated with lower prevalence and improved prognosis of CKD, while no association was observed for unfavorable DI-GM, suggesting that dietary patterns favorable to gut microbiota health play a key role in CKD protection.

Our findings demonstrate a clear association between higher beneficial DI-GM components and lower CKD risk, whereas no significant association was observed for unfavorable DI-GM components. This suggests that promoting beneficial dietary components may be more effective in preventing CKD progression than merely reducing harmful ones. Studies indicate that dietary fiber, antioxidants, and fermented foods improve gut health by promoting the production of beneficial short-chain fatty acids, which exert anti-inflammatory effects and help maintain intestinal barrier integrity, while reducing gut-derived uremic toxins that exacerbate CKD progression (Ranganathan and Anteyi, 2022; Zhang et al., 2024). Beneficial DI-GM components, such as a diet high in fermented foods, have been shown to steadily increase microbiota diversity and reduce inflammatory markers, thereby protecting the intestinal barrier (Budau et al., 2024). In contrast, Western-type diets damage the intestinal barrier by disrupting gut microbiota composition and causing inflammation. Excessive consumption of unfavorable DI-GM components, such as refined grains (a staple of the Western diet), leads to hyperglycemia, which is associated with gut dysbiosis and contributes indirectly to CKD progression (Shehab-Eldin et al., 2009). Furthermore, a Mendelian randomization study by Feng et al. (Feng et al., 2024) demonstrated causal links between specific bacterial genera, including Bifidobacterium and Akkermansia, and renal outcomes like nephrotic syndrome and CKD. These findings support our conclusion that a higher DI-GM, which includes beneficial dietary elements promoting these bacteria, may be linked to a reduced risk of CKD.

Notably, coffee and fiber consumption had the most significant impact on the prevalence and prognosis of CKD. A meta-analysis by Srithongkul et al. (Srithongkul and Ungprasert, 2020), including 25,849 participants, showed that coffee consumption was associated with a lower risk of incident CKD, with a pooled risk ratio of 0.87 (95 % CI: 0.81–0.95). Furthermore, a prospective cohort study with 359,906 participants from the UK Biobank demonstrated an inverse association between coffee consumption and incident CKD, with females benefiting more from this association (Tang et al., 2022). Coffee contains bioactive compounds, such as polyphenols, known for their antioxidant and anti-inflammatory properties. These compounds may help mitigate the oxidative stress and inflammation that are critical drivers of CKD progression. Although caffeine, a major component of coffee, temporarily raises blood pressure, long-term consumption has been associated with lower systemic blood pressure, which is crucial for preventing CKD (Jhee et al., 2018). Furthermore, studies have identified specific metabolites in serum related to coffee intake. For instance, glycochenodeoxycholate, a lipid involved in bile acid metabolism, has been linked to kidney health and coffee consumption (He et al., 2021). Metabolites related to benzoate metabolism, such as *O*-methylcatechol sulfate and 3-methylcatechol sulfate, were found to be positively associated with coffee consumption and incident CKD (He et al., 2021). Increased fiber intake has been linked to a reduced risk of developing CKD and improved kidney outcomes in patients with existing disease (Heo et al., 2023). Fermentable fibers, such as inulin and oligosaccharides, promote the growth of saccharolytic bacteria, which produce beneficial metabolites, including short-chain fatty acids. These fatty acids play a role in modulating the immune system by promoting regulatory T cells and suppressing pro-inflammatory T helper 17 cells (Serrano et al., 2022). A study in CKD patients found that higher fiber intake was associated with lower serum creatinine and urea levels (Su et al., 2022). Additionally, fiber intake may positively impact metabolic parameters, including blood pressure and lipid profiles, further supporting kidney health. Interestingly, we found that whole grains had a beneficial effect on CKD prognosis independent of other components. Studies have shown that increasing fiber intake, particularly from fruits, vegetables, legumes, and whole grains, can improve kidney function and reduce the risk of disease progression (Heo et al., 2023; Su et al., 2022). Our findings align with previous research, providing a more comprehensive understanding of the role of diet in CKD outcomes.

The connection between the gut microbiome and CKD progression can be explained by the impact of gut dysbiosis and its metabolites on kidney function. Dysbiosis—characterized by reduced gut microbial diversity and an overabundance of pathogenic microbes—leads to harmful metabolite production, such as indoxyl sulfate and p-cresyl sulfate, both of which are nephrotoxic and contribute to kidney inflammation and fibrosis (Huang and Chen, 2024; Johnson-Martínez et al., 2024). Probiotic and synbiotic interventions have shown promise as adjuvant therapies for CKD. Studies like the SYNERGY II trial have demonstrated the potential of synbiotics to enhance gut microbiota diversity, reduce nephrotoxic metabolites, and slow CKD progression (McFarlane et al., 2021). Recently, Liu et al. (Liu et al., 2024) demonstrated that hyperuricemia, a common CKD comorbidity, exacerbates renal injury by inducing oxidative stress and mitochondrial dysfunction, partly mediated through gut microbiota changes. In hyperuricemic models, significant changes in the gut microbiome were observed, including alterations in the abundance of Akkermansia and Lachnospiraceae, both of which are linked to important metabolic pathways such as amino acid and biotin metabolism. This imbalance led to increased oxidative stress in kidney tissues, evidenced by elevated malondialdehyde levels, and induced mitophagy, a compensatory mechanism that may further deplete renal function when sustained chronically (Liu et al., 2024). These findings indicate that mitigating hyperuricemia through dietary interventions enhancing DI-GM could help restore metabolic balance and reduce oxidative damage in CKD patients. Moreover, reducing foods high in purines may complement DI-GM's positive effects on gut and kidney health. Another critical mechanism involves trimethylamine N-oxide, a gut microbial metabolite derived from dietary choline, lecithin, and l-carnitine, which has been implicated in renal fibrosis and CKD progression (Lee et al., 2024). Recent studies have shown that elevated trimethylamine N-oxide levels are significantly associated with an increased risk of acute kidney injury progressing to CKD. Modulation of the gut microbiome through antibiotic-induced microbiota depletion was found to reduce trimethylamine N-oxide levels, thereby attenuating oxidative stress, inflammation, and kidney fibrosis in animal models (Lee et al., 2024).

This study has several strengths. First, the use of a nationally representative sample and application of survey weights ensure the generalizability of findings. Second, this is the first study to assess the association between DI-GM and CKD, emphasizing the importance of beneficial DI-GM components. Third, we explored not only the association with CKD prevalence but also DI-GM's impact on CKD prognosis. Lastly, we identified the independent association between specific components of DI-GM and CKD.

However, this study has several limitations. First, the cross-sectional design of NHANES precludes establishing causal relationships (Wu et al., 2024). Second, despite adjustments for confounding factors, residual confounders such as medication history may remain. Third, we did not adjust for biochemical indicators such as blood and urine measures, which may have influenced gut microbial imbalances and kidney damage, potentially affecting the results. Future research should focus on longitudinal studies to establish causal relationships and further explore the mechanisms through which beneficial dietary patterns exert protective effects on renal function.

## 5 Conclusion

In conclusion, this study reveal a linear negative association between DI-GM and both CKD prevalence and its prognosis, suggesting that promoting beneficial dietary components—rather than merely reducing harmful ones—may be more effective in preventing CKD onset and progression. Coffee and fiber were the most significant contributors to the prevalence of CKD, while coffee, fiber, and whole grains had the greatest impact on its prognosis. The results underscore the potential of dietary interventions targeting gut microbiota health to serve as a critical element in CKD management strategies.

## CRediT authorship contribution statement

**Xuanzhen Zhou:** Writing – review & editing, Writing – original draft, Software, Methodology, Investigation, Formal analysis, Conceptualization. **Chengxiao Jiang:** Writing – review & editing, Writing – original draft, Methodology. **Baiyang Song:** Writing – review & editing, Validation. **Shuben Sun:** Writing – review & editing, Supervision, Project administration. **Zejun Yan:** Writing – review & editing, Project administration, Methodology, Conceptualization.

## Ethical approval and consent to participate

The study protocol for the U.S. NHANES was approved by the National Center for Health Statistics Research ethics review board (Protocol #2005–06, #2011–17, #2018–01). All participants provided written informed consent. Institutional review board approval was waived for this analysis because of the publicly available and deidentified data.

## Funding

This work was supported by the Ningbo Top Medical and Health Research Program [grant numbers 2022020203]; the Ningbo Clinical Research Center for Urological Disease [grant numbers 2019A21001]; and the Ningbo Technological Innovation 2025 Projects [grant numbers 2021Z070].

## Declaration of competing interest

The authors declare that they have no known competing financial interests or personal relationships that could have appeared to influence the work reported in this paper.

## Data Availability

The datasets generated during and/or analysed during the current study are available in the National Health and Nutrition Examination Survey, https://www.cdc.gov/nchs/nhanes/index.htm.

## Appendix A Supplementary data

Supplementary material 1 Supplementary Table S1-S3 Supplementary material 1

Supplementary material 2 Ethical Exemption Statement Supplementary material 2
