# Supplementary material for: Association between dietary index for gut microbiota and chronic kidney disease: A cross-sectional study from U.S. population
Source: Prev Med Rep. 2025 Apr 8;53:103060. doi: 10.1016/j.pmedr.2025.103060 (PMC12013330; doi:10.1016/j.pmedr.2025.103060)
Supplement: Supplementary material 1 — Supplementary Table S1-S3 [file mmc2.docx]

Supplementary Tables

| Supplementary Table S1. Detailed information on the classification of covariates | |
| --- | --- |
| Covariates | Definition of classification |
| Household income | Family poverty-to-income ratio: low (≤ 1), middle (1–4), and high (≥ 4). |
| Smoking status | Never (smoked less than 100 cigarettes in life), former (smoking >100 cigarettes in life but does not smoke now), and now (smoking ≥ 1 cigarette every day). |
| Drinking status | Never (< 12 drinks in lifetime), former (consumed ≥ 12 drinks in a single year/ lifetime but did not drink last year), and now (≥ 12 drinks over the past year). |
| Physical activity | Defined as having done at least 10 min of moderate or vigorous in a typical week. |
| Hypertension | Defined as systolic blood pressure ≥ 140 mmHg and/or diastolic blood pressure ≥ 90 mmHg, self-reported hypertension, or receiving anti-hypertensive medication. |
| Diabetes mellitus | Defined as glycated hemoglobin A1c ≥ 6.5%, fasting blood glucose ≥ 7.0 mmol/L, two-hour oral glucose tolerance test ≥ 11.1 mmol/L, self-reported diabetes, or use of insulin or hypoglycemic medication. |
| Hyperlipidemia | Defined as triglycerides ≥ 150 mg/dL, total cholesterol ≥ 200 mg/dL, low-density lipoprotein cholesterol ≥ 130mg/dL, HDL-C ≤ 40mg/dL for males and ≤ 50mg/ dL for females, or receiving lipid-lowering medications. |

| Supplementary Table S2. Association between dietary index for gut microbiota and the key markers of kidney function in U.S. adults from NHANES 2007–2020 | | | |
| --- | --- | --- | --- |
|  | Model 1 | Model 2 | Model 3 |
|  | *β* (95%CI) | *β* (95%CI) | *β* (95%CI) |
| UACR |  |  |  |
| DI-GM | -2.26 (-3.91, -0.61) | -2.52 (-4.27, -0.76) | -0.98 (-2.65, 0.70) |
| DI-GM group |  |  |  |
| 0–3 | 1 (ref.) | 1 (ref.) | 1 (ref.) |
| 4 | -5.14 (-13.63, 3.34) | -4.91 (-13.51, 3.69) | -2.40 (-11.25, 6.45) |
| 5 | -6.88 (-15.50, 1.74) | -7.29 (-15.90, 1.32) | -2.27 (-10.90, 6.36) |
| ≥6 | -10.75 (-18.01, -3.49) | -11.74 (-19.42, -4.05) | -4.95 (-12.50, 2.60) |
| *P* for trend | 0.584 | 0.635 | 0.625 |
| Beneficial DI-GM | -3.80 (-5.77, -1.83) | -3.50 (-5.53, -1.47)^*^ | -1.78 (-3.75, 0.18)^*^ |
| Unfavorable DI-GM | 0.71 (-2.00, 3.42) | -0.91 (-3.77, 1.96)^#^ | 0.33 (-2.48, 3.13)^#^ |
| eGFR |  |  |  |
| DI-GM | -0.88 (-1.11,-0.65) | 0.22 (0.08, 0.37) | 0.27 (0.13, 0.41) |
| DI-GM group |  |  |  |
| 0–3 | 1 (ref.) | 1 (ref.) | 1 (ref.) |
| 4 | 0.11 (-0.69, 0.90) | 0.29 (-0.34, 0.92) | 0.31 (-0.31, 0.93) |
| 5 | -1.95 (-2.87,-1.02) | 0.06 (-0.66, 0.77) | 0.13 (-0.56, 0.82) |
| ≥6 | -3.54 (-4.54,-2.55) | 0.85 (0.22, 1.49) | 1.02 (0.41, 1.63) |
| *P* for trend | 0.15 | 0.112 | 0.1 |
| Beneficial DI-GM | -0.75 (-1.01,-0.60) | 0.45 (0.28, 0.62)^*^ | 0.51 (0.34, 0.68)^*^ |
| Unfavorable DI-GM | -0.88 (-1.21,-0.55) | -0.15 (-0.37, 0.08)^#^ | 0.33 (-2.48, 3.13)^#^ |
| All estimates were obtained from complex survey designs, *p* for trend was obtained from multivariable logistic regression analysis.  Model 1: unadjusted  Model 2: adjusted for age, sex, race/ethnicity  Model 3: adjusted for age, sex, race/ethnicity, marital status, household income, education level, body mass index, smoking status, drinking status, physical activity, hypertension, diabetes mellitus, hyperlipidemia  ^*^Further adjusted for unfavorable DI-GM  ^#^Further adjusted for beneficial DI-GM  DI-GM dietary index for gut microbiota. UACR urinary albumin-to-creatinine ratio. eGFR estimate glomerular filtration rate. | | | |

| Supplementary Table S3. Association between specific components of dietary index for gut microbiota and chronic kidney disease in U.S. adults from NHANES 2007–2020 | | | | |
| --- | --- | --- | --- | --- |
| DI-GM Components (score) | CKD Prevalence | | CKD Prognosis at very high risk | |
|  | Model 3 | Model 3* | Model 3 | Model 3* |
|  | OR (95%CI) | OR (95%CI) | OR (95%CI) | OR (95%CI) |
| Avocados |  |  |  |  |
| 1 vs. 0 | 1.20 (0.90, 1.61) | 1.31 (0.98, 1.75) | 0.59 (0.20, 1.69) | 0.73 (0.24, 2.25) |
| Broccoli |  |  |  |  |
| 1 vs. 0 | 0.92 (0.75, 1.12) | 0.94 (0.77, 1.16) | 1.01 (0.6, 1.71) | 1.14 (0.67, 1.93) |
| Chickpeas |  |  |  |  |
| 1 vs. 0 | 0.75 (0.44, 1.26) | 0.81 (0.49, 1.38) | 0.123 (0.02, 0.95) | 0.16 (0.02, 1.27) |
| Coffee |  |  |  |  |
| 1 vs. 0 | 0.83 (0.75, 0.92) | 0.83 (0.75, 0.92) | 0.71 (0.56, 0.91) | 0.73 (0.57, 0.93) |
| Cranberries |  |  |  |  |
| 1 vs. 0 | 1.20 (0.97, 1.47) | 1.21 (0.99, 1.49) | 0.98 (0.64, 1.50) | 1.11 (0.73, 1.70) |
| Fermented dairy |  |  |  |  |
| 1 vs. 0 | 0.91 (0.83, 1.01) | 0.94 (0.85, 1.05) | 0.86 (0.69, 1.08) | 0.91 (0.71, 1.18) |
| Fiber |  |  |  |  |
| 1 vs. 0 | 0.83 (0.75, 0.91) | 0.83 (0.75, 0.91) | 0.52 (0.43, 0.64) | 0.58 (0.46, 0.74) |
| Green tea |  |  |  |  |
| 1 vs. 0 | 0.96 (0.83, 1.10) | 0.96 (0.83, 1.10) | 0.85 (0.60, 1.20) | 0.85 (0.59, 1.22) |
| Soybean |  |  |  |  |
| 1 vs. 0 | 0.90 (0.77, 1.05) | 0.93 (0.8, 1.09) | 0.96 (0.66, 1.39) | 1.12 (0.77, 1.62) |
| Whole grains |  |  |  |  |
| 1 vs. 0 | 0.96 (0.86, 1.06) | 1.02 (0.92, 1.14) | 0.60 (0.47, 0.76) | 0.72 (0.56, 0.94) |
| Refined grains |  |  |  |  |
| 1 vs. 0 | 1.09 (1.00, 1.20) | 1.04 (0.93, 1.15) | 1.10 (0.91, 1.33) | 0.98 (0.81, 1.2) |
| Processed meat |  |  |  |  |
| 1 vs. 0 | 1.09 (0.98, 1.22) | 1.11 (0.98, 1.25) | 1.23 (0.97, 1.56) | 1.22 (0.93, 1.61) |
| Red meat |  |  |  |  |
| 1 vs. 0 | 0.97 (0.88, 1.07) | 0.93 (0.83, 1.03) | 1.01 (0.80, 1.28) | 0.95 (0.73, 1.23) |
| High-fat diet |  |  |  |  |
| 1 vs. 0 | 1.06 (0.95, 1.19) | 1.05 (0.94, 1.18) | 0.94 (0.72, 1.23) | 0.95 (0.73, 1.24) |
| Model 3: adjusted for age, sex, race/ethnicity, marital status, household income, education level, body mass index, smoking status, drinking status, physical activity, hypertension, diabetes mellitus, hyperlipidemia.  Model 3*: further adjusted for all components of DI-GM based on Model 3.  DI-GM dietary index for gut microbiota. | | | | |
