# Supplementary material for: Association between dietary index for gut microbiota and chronic kidney disease: A cross-sectional study from U.S. population
Source: Prev Med Rep. 2025 Apr 8;53:103060. doi: 10.1016/j.pmedr.2025.103060 (PMC12013330; doi:10.1016/j.pmedr.2025.103060)
Supplement: Supplementary material 2 — Ethical Exemption Statement [file mmc3.pdf]

伦理证明

preventive medicine reports 杂志社：

兹证明本院投稿贵刊的论文《Association between dietary index for gut microbiota and chronic kidney disease:A cross-sectional study from U.S. population》，是由周轩震、孙树本、严泽军等基于美国国家健康与营养调查（NHANES）数据库所撰写。未涉及患者的生物医学信息及生物样本等实验性研究，为利用合法获得的公开数据进行二手数据分析。其中，美国国家卫生统计中心的研究伦理审查委员会批准了 NHANES 的研究。所有提供书面知情同意书的参与者和研究程序均由国家卫生统计中心的研究伦理审查委员会批准。伦理审查委员会批准的详细信息可在下列网址(https://www.cdc.gov/nchs/nhanes/.)找到。

综上，根据《涉及人的生命科学和医学研究伦理审查办法》，该论文设计的研究内容可以免除伦理审查。特此说明。

宁波大学附属第一医院医学伦理委员会

2025-04-02

情况属实

第一作者签名 周轩震

通讯作者签名 孙树本

科主任签名 严泽军
